# New Symptoms of Duodenal Ulcer

**Published:** 1916-04

**Authors:** 


**New Symptoms of Duodenal Ulcer.** Heinrich Stern, Arch. of Diag., No. 4, 1915, states that, to prevent epigastric pain, the patient assumes the standing posture in preference to sitting or stretches out in a long slant, whether in a chair or bed. He may rest on the back or left side but not on the right. This sign is not present in gastric ulcer (?Ed.). There is none of the tendency to doubling up manifested in appendix inflammation, gall stones, colic, etc.

